# Special Diagnosis and Homœopathic Treatment of Diseases

**Published:** 1893-04

**Authors:** 


### Special Diagnosis and Homœopathic Treatment of Diseases. By T. de Suzzara Verdi, M. D. Philadelphia: Bœricke & Tafel.

By the request of the many readers of the author's previous work, entitled *Maternity*, the author "has enlarged the scope of that special work so as to cover all diseases," making it a work on general domestic practice. The object of the book is for treatment in emergencies and in the absence of a physician. It treats mostly of diseases in acute forms which demand immediate attention. It is divided into nine parts, and each part treats of a different disease. The language is plain, and technical terms, when employed, are explained. A "doctor book" may be of great value in a house, but may be made a source of evil—by using it too frequently where the calling in of a good physician would be of much greater benefit. Homœopathic physicians, who have been called into sudden cases of illness, and have found their opportunity for making a clever prescription of the simillimum spoiled by the ill-judged administration of remedies by a member of the household who made a hasty consultation of one of these books of domestic practice, will hear of this new candidate for home favor with dismay.

